# ‘Some’ Effects of Age, Task, Task Content and Working Memory on Scalar Implicature Processing

**DOI:** 10.5334/pb.ax

**Published:** 2014-09-16

**Authors:** Leen Janssens, Iris Fabry, Walter Schaeken

**Affiliations:** 1 Laboratory of Experimental Psychology, KU, Leuven (University of Leuven), Belgium

**Keywords:** scalar implicature, working memory, task, task content, age

## Abstract

In three experiments, we investigated the effect of age, task, task content and working memory (WM) on scalar implicature processing. We found that three-year-olds still often interpret the scalar term ‘some’ logically (some being compatible with all), but five-year-olds and especially seven-year-olds are highly competent pragmatic reasoners. Additionally we found that not only the nature of the task but also the specific task content influences the number of pragmatic answers: an Action-Based-Task (ABT) leads to more pragmatic answers than a metalinguistic Truth-Value Judgment Task (TVJT) that, in turn, leads to more pragmatic answers than a different TVJT that includes more cognitive content. Finally, we found no effect of WM in both five-year-olds and seven-year-olds. Children with a high WM capacity did not provide significantly more pragmatic answers than children with a low WM capacity.

## Introduction

People communicate with each other to express what they feel, think, want, etc. Although this seems to happen effortlessly and automatically, the communication process involves more than just decoding a message between a messenger and a receiver. Apart from the literal meaning of a sentence, a speaker can convey a lot more by communicating an implicit meaning that is not explicitly expressed. The first systematic attempt to explain how these inferences are derived, belongs to Paul Grice. He offered a comprehensive framework of the mechanics of inferential communication (Grice, 1975). According to Grice, communication is a cooperative enterprise between people, governed by certain relational expectations about how a conversational exchange should be conducted. These relational expectations are called ‘maxims’ and Grice proposed four of these maxims: the Maxim of Quantity, the Maxim of Quality, the Maxim of Relation and the Maxim of Manner. These maxims respectively imply that interlocutors are always expected to offer contributions which are informative, truthful, relevant to the goals of the conversation and appropriately phrased. Grice introduced the term ‘implicature’, which refers to the meaning that is implied by the speaker but not explicitly stated.

Considerable experimental research has been devoted to *scalar implicatures*, i.e. implicatures based on the existence of ordered terms on a scale of informativity (e.g., <all, most, many, some>, <always, often, sometimes>, etc.). A scalar expression such as *some* can be interpreted in two different ways: either pragmatically (‘some but not all’) or logically (‘some and perhaps all’). Whenever a weaker term (e.g., the quantifier *some*) is used, the general consensus is that a stronger term from the same scale (e.g., *all*) does not hold because the speaker did not use the stronger term. If the stronger term was applicable then the speaker would have been underinformative.

Experimental investigations into children’s interpretation of scalar terms have concluded that preschool children are often insensitive to scalar implicatures in tasks involving language comprehension (Chierchia, Crain, Guasti, Gualmini, & Meroni, 2001; Noveck, 2001). In these studies, children seemed to attend only to the logical/semantic meaning of the scalar terms, even though they were shown to be linguistically competent. For example, Noveck (2001) found that 89 per cent of the seven-to-eight-year olds in his study agreed with statements such as ‘*Some* giraffes have long necks’. Noveck (2001) concluded that ‘younger, albeit competent reasoners, initially treat a relatively weak term logically before becoming aware of its pragmatic potential’, and that, in this respect, ‘children are more logical than adults’ (Noveck 2001: 165).

The availability of cognitive resources is often used to explain this typically found pragmatic delay in children. As suggested by Noveck (2001), a plausible explanation for this delay is that inferring scalar implicatures requires effort and that children have less cognitive resources available than adults. There are two major theories with opposite predictions regarding this issue. According to the default theories (e.g., neo-Gricean theories; e.g., Levinson, 2000), implicature production happens automatically and only its inhibition demands processing costs. Contextual theories (e.g., Relevance Theory; Sperber & Wilson, 1995) in contrast, suggest that an implicature will only be produced if it is relevant in the context and they state that this production requires additional processing costs. Evidence in favor of Relevance Theory, regarding scalar implicatures, has been presented among others by Noveck and Posada (2003). Their experiments indicated that pragmatic answers require more time than logical answers. Assuming that longer time is associated with more processing costs, this provides indirect evidence for Relevance Theory.

In contrast to research showing that children initially reason logically, there is also substantial experimental evidence that children are not incapable of drawing scalar inferences and that they are aware of the pragmatic potential of scalar expressions. In these kinds of studies, the prime interest is to discover what conditions facilitate implicature production for children. A key factor seems to be the nature of the task. For instance, Foppolo, Guasti, and Chierchia (2004) conducted experiments concerning the quantitative scale <all, some> using two different tasks: a Truth-Value Judgment Task (TVJT) (Crain & Thornton, 1998), in which participants had to decide whether (underinformative) statements were true or false, and a Felicity Judgment Task (FJT) (Chierchia et al., 2001). In the FJT, participants were presented with a pair of utterances with the same truth-value but different levels of appropriateness and were asked to choose the most felicitous description. When five-year-olds completed the FJT, the number of pragmatic responses was 95 per cent while the number of pragmatic responses on the TVJT was only 50 per cent.

Pouscoulous, Noveck, Politzer, and Bastide (2007) also examined the role of the nature of the task. In their first experiment, they replicated earlier findings showing that nine-year-olds were more likely than adults to consider as true statements such as ‘*some* turtles are in the boxes’ (uttered when *all* turtles are in the boxes) in a TVJT. In their second experiment, they presented an Action-Based Task (ABT), in which participants did not have to give a metalinguistic evaluation of statements but had to respond by performing an action. Children were presented with five boxes and five tokens. They were asked to adapt the situation to make it compatible with a statement. For example, if they were told ‘I would like *all* the boxes to contain a token’ and two of the five boxes already contained a token, they were expected to put a token in every empty box. The results showed that, when children were asked to perform an action rather than give a metalinguistic truth evaluation, the number of derived implicatures in children increased.

In the present study we build on these experiments by Pouscoulous et al. (2007). In Experiment 1, we compare pragmatic processing between a group of three-year-old children and a group of five-year-old children. To our knowledge, no scalar implicature research has been done with children under the age of four. We will investigate whether three-year-olds are already capable of understanding scalar implicatures and whether their performance differs from the five-year-olds. Second, we wonder if there will be an influence of the nature of the task.

In Experiment 2, a group of only five-year-olds will perform the same tasks as in Experiment 1, but additionally their working memory (WM) capacity will be measured in order to test the hypothesis that children with a high WM span will provide more pragmatic answers than children with a low WM span. Again, the nature of the task is also investigated as a possible influential factor in pragmatic processing.

Experiment 3 involves a group of seven-year-old children. The WM hypothesis as well as the hypothesis regarding the nature of the task are tested again in this slightly older age group. Moreover, an additional TVJT is presented that only differs from the other TVJT in that it requires world-knowledge to solve the items. We wondered whether there would be an effect of the (cognitive) content on the pragmatic response rate.

## Experiment 1

In our first experiment, both three-year-olds and five-year-olds were tested. Our primary goal was to test such young children’s pragmatic competence by means of two different implicature tasks. It has been shown that the nature of the task has an influence on the number of pragmatic answers in children (e.g., Pouscoulous et al., 2007) so we included two different tasks based on Pouscoulous et al. (2007). We made two important changes to the Pouscoulous et al. (2007) study. First, we presented the same group of children with both a TVJT and an ABT: manipulating the nature of the task within subjects allows direct comparison between the two tasks. Second, there was an important difference in content between the ABT and TVJT used by Pouscoulous et al. (2007). Whereas the ABT in Pouscoulous et al. (2007) only used tokens and boxes, in the TVJT, the children were presented with three types of animals that remained in front of them throughout the task. For each statement, they had to focus on one type of animal and ignore the other animals. Since the statements were randomly ordered, they constantly had to switch their attention between the three types, which placed greater demands on information processing than in the ABT. To remedy this problem, we made the two tasks more similar in design by using the same scenario’s with marbles and boxes in both tasks.

We had two hypotheses. First, we expected to find an age effect: we expected the five-year-olds to be more pragmatic on the critical items than the three-year-olds. Second, we expected to find an effect of the nature of the task. We expected the ABT to be easier and therefore to lead to more pragmatic answers than the TVJT.

### Method

#### Participants

The sample comprised 20 three-year-olds (14 boys and six girls) between the ages of 36 and 52 months with a mean age of 44 months (*SD*=4.7) and 23 five-year-olds (12 boys and eleven girls) between the ages of 55 and 71 months with a mean age of 62 months (*SD*=5.04). They were recruited from a primary school in Belgium (Sint-Annaschool, Duisburg). All were native Dutch speakers, including some bilingual children.

#### Action-Based Task (ABT)

The ABT consisted of three scenarios, each involving five plastic boxes and five marbles. In the ‘All-scenario’, all five boxes contained a marble. In the ‘None-scenario’, all the boxes were empty. In the ‘Subset-scenario’, two boxes contained a marble. In each scenario, a puppet, handled by the experimenter, was used to utter the same four requests: ‘I would like *all* the boxes to contain a marble’ (*“Ik zou willen dat er in alle dozen een knikker zit”*), ‘I would like *some* boxes to contain a marble’ (*“Ik zou willen dat er in sommige dozen een knikker zit”*), ‘I would like *none* of the boxes to contain a marble’ (*“Ik zou willen dat er in geen van de dozen een knikker zit”*) and ‘I would like *some* boxes *not* to contain a marble’ (*“Ik zou willen dat er in sommige dozen geen knikker zit”*). This amounted to a total of 12 requests. The participants were instructed to make changes to the scenario to comply with the puppet’s requests. For example, if the puppet said ‘I would like *all* the boxes to contain a marble’ in the ‘Subset-scenario’, the child was expected to put a marble in the three empty boxes.

There were two critical situations and ten control statements. The first critical statement occurred in the ‘All-scenario’ when the puppet stated ‘I would like *some* boxes to contain a marble’. If the child interprets *some* logically, he or she will make no changes to the scenario. However, if the child grasps the implicature, he or she will take at least one and maximum four of the marbles away. The second critical statement occurred in the ‘None-scenario’ when the puppet uttered the statement ‘I would like *some* boxes *not* to contain a marble’. In this case, if the child interprets the statement logically, no action should be taken. A pragmatic interpretation on the other hand would require an action (adding at least one and maximum four marbles to the boxes).

For the ten control statements, there was a distinction possible between pragmatic and logical interpretations, only for the *some (not)* sentences. For example: when the request ‘I would like *some* boxes to contain a marble’ is uttered in the ‘None-scenario’, a wrong answer would be to change nothing, a pragmatic answer would be to add one to four marbles and a logical answer would be to put a marble in every box. All other control sentences were either right or wrong; E.g., ‘I would like *all* the boxes to contain a marble’ in the ‘None-scenario’. In this case the child is expected to put a marble in all five empty boxes. All other actions would be wrong.

#### Truth-Value Judgment Task (TVJT)

The children were presented with five boxes and five marbles in the three same scenarios as in the ABT. In each scenario, a puppet made the same four statements (amounting to a total of 12 sentences): ‘*All* the marbles are in the boxes’ (*“Alle knikkers zitten in de dozen”*), ‘*Some* marbles are in the boxes’ (*“Sommige knikkers zitten in de dozen”*), ‘*None* of the marbles are in the boxes’ (*“Geen van de knikkers zit in een doos”*) and ‘*Some* marbles are *not* in the boxes’ (*“Sommige knikkers zitten niet in de dozen”*). After each statement, participants had to decide whether the statement was true or false. The two critical statements were ‘*Some* marbles are in the boxes’ in the ‘All-scenario’ and ‘*Some* marbles are *not* in the boxes’ in the ‘None-scenario’. In both cases, ‘true’ would be the logical answer, whereas ‘false’ would be the pragmatic answer.

The other ten statements were control statements (e.g., ‘*Some* marbles are in the boxes’ in the ‘Subset-scenario’). These statements could only be answered right or wrong, in contrast to the control statements of the ABT.

#### Procedure

Each participant was interviewed individually for about 20 minutes. For both age groups, the order of the two tasks was randomized, so that half of the participants started with the TVJT and the other half with the ABT. In both tasks, the experimenter used a puppet called Knorrie. In the TVJT, the children were informed that the puppet sometimes says things that are correct and sometimes says things that are wrong. In the ABT, the children were told that the puppet would give requests regarding the boxes and the marbles and that they would either have to remove marbles, add marbles, or make no changes. Before the start of the experiment, the children were given three practice questions in the ABT. These questions were very similar to the experimental sentences but employed numbers instead of quantifiers. The three training questions were: ‘I would like two boxes to contain a marble’, when only one box contained a marble, ‘I would like three boxes to contain a marble’, when three boxes contained a marble and ‘I would like two boxes to contain a marble’, when three boxes contained a marble. These training questions were constructed so that the participants had to add marbles, change nothing and remove marbles. This way, they got acquainted with all types of actions they would have to perform during the experiment. If the children made errors on these training questions, the experimenter corrected them and explained their mistakes.

### Results

In a first analysis, we controlled whether we could exclude an effect of order. We analyzed if the order of the two implicature tasks influenced the number of pragmatic answers. For example, it could be that being presented with the ABT first facilitates pragmatic responding on the TVJT or vice versa. We performed an ANOVA on both the TVJT and the ABT. There was no significant effect of order (*F*(1, 34)=0.128; *p*=.72), nor a significant interaction effect between age group and order (*F*(1, 34)=0.279; *p*=.60) on pragmatic responding for the TVJT. Likewise, there was no significant effect or order (*F*(1, 34)=0.688; *p*=.41), nor a significant interaction effect with age group (*F*(1, 34)=0.59; *p*=.45) for the ABT.

Our first hypothesis concerned an effect of age. We expected five-year-olds to be more pragmatic than three-year-olds. Our second hypothesis concerned an effect of the nature of the task. We expected that the ABT would lead to more pragmatic answers than the TVJT.

With regard to our first hypothesis, we first compared the two age groups concerning the number of pragmatic answers they provided. However, in our analyses of the critical items of the ABT, five three-year-olds were excluded. These children didn’t provide logical or pragmatic answers on the critical items of the ABT, but simply wrong answers (i.e. taking all marbles away when they were asked ‘I would like *some* boxes to contain a marble’ in the ‘All-scenario’ and/or putting a marble in all the boxes when they were asked ‘I would like *some* boxes *not* to contain a marble’ in the ‘None-scenario’). These five children were included in all other analyses.

We found that the three-year-olds were significantly less pragmatic than the five-year-olds on both the ABT (Mann-Whitney U Test, *n_1_*=15, *n_2_*=23, *U*=98.5, *Z*=-2.43, *p*=.008) and the TVJT (Mann-Whitney U Test, *n_1_*=20, *n_2_*=23, *U*=119, *Z*=-2.94, *p*=.002). The three-year-olds provided 46.7 per cent (ABT) and 45.0 per cent (TVJT) pragmatic answers compared to 80.4 per cent (ABT) and 76.1 per cent (TVJT) for the five-year-olds.

In the analyses described above, the affirmative (i.e. *some*) and the negative (i.e. *some not*) critical item were taken together to compute the pragmatic response rate. When we look at those items separately for the TVJT, we found that there was a significant difference between the two age groups for the affirmative item (Mann-Whitney U Test, *n_1_*=20, *n_2_*=23, *U*=96.0, *Z*=-3.77, *p*<.001). The three-year-olds only provided 20 per cent pragmatic answers on this item, compared to 78.3 per cent for the five-year-olds. However, the difference between the two age groups was not significant for the negative item of the TVJT (Mann-Whitney U Test, *n_1_*=20, *n_2_*=23, *U*=221.0, *Z*=-0.28, *p*=.78). On this negative item, the three-year-olds and the five-year-olds respectively provided 70 per cent and 73.9 per cent pragmatic answers. The very low percentage of pragmatic answers on the affirmative item for the youngest children therefore causes the significant difference between the positive and the negative critical item of the TVJT (*t*(42)=-2.15; *p*=.037) for the whole sample of children (51.2 per cent pragmatic answers on the affirmative item compared to 72.1 per cent on the negative item).

When we look at the two critical items of the ABT separately, we found that there was a significant difference between the age groups on both the affirmative item (Mann-Whitney U Test, *n_1_*=17, *n_2_*=23, *U*=134.5, *Z*=-2.02, *p*=.043) and the negative item (Mann-Whitney U Test, *n_1_*=16, *n_2_*=23, *U*=112.5, *Z*=-2.50, *p*=.012). The youngest and oldest children respectively provided 47.1 per cent and 78.3 per cent pragmatic answers on the affirmative item and 43.8 per cent and 82.6 per cent pragmatic answers on the negative item. However, there was no significant difference in pragmatic response rate between the affirmative and the negative item of the ABT for the whole sample (*t*(37)=0.298; *p*=.77).

Apart from the difference in pragmatic responding, we also found that the older children provided more correct answers on the control items than the younger children on both tasks (ABT: 97.4 per cent vs 88.5 per cent correct answers; Mann-Whitney U Test, *n_1_*=20, *n_2_*=23, *U*=124.5, *Z*=-2.98, *p*=.002; TVJT: 93.9 per cent vs 84.5 per cent correct answers; Mann-Whitney U Test, *n_1_*=20, *n_2_*=23, *U*=111.5, *Z*=-3.07, *p*=.001).

With regard to our second hypothesis, we analyzed the two age groups combined. We found that the ABT was significantly easier than the TVJT since it led to more correct answers on the control sentences (93.3 per cent vs 89.5 per cent correct answers; Wilcoxon Signed Ranks test, *n*=26; *T*=88.5; *p*=.01). The easier ABT also led to more pragmatic answers (67.1 per cent) than the TVJT (61.5 per cent) but this difference was not significant (Wilcoxon Signed Ranks test, *n*=18; *T*=106.0; *p*=.17).

When we look at the difference between the two tasks for the two age groups separately, we only found a significant difference between the control sentences of the ABT and the TVJT for the five-year-olds (Wilcoxon Signed Ranks test, *n*=13; *T*=23; *p*=.049) and a marginally significant difference for the three-year-olds (Wilcoxon Signed Ranks test, *n*=13; *T*=23; *p*=.051).

The ten control sentences of the ABT included four requests (the requests with *some (not)*) that could be answered in three different ways; either wrong (e.g., taking all marbles away when the request was ‘I would like *some* boxes to contain a marble’), pragmatically (e.g., placing one to four marbles in the boxes when the request was *some*), or logical (e.g., placing a marble in all the boxes when *some* was requested). In our analyses above, we scored both the pragmatic and the logical answer as correct. However, when we look at the different types of answers separately, we find a significant difference between our two age groups. The three-year-olds provided 45.0 per cent logical and 45.0 per cent pragmatic answers on these sentences compared to 7.6 per cent logical and 90.2 per cent pragmatic answers for the five-year-olds (*X*^2^=41.1, *df*=2, *p*<.001).

### Discussion

Our results confirmed that there is an effect of age in pragmatic competence. Three-year-olds still often provide a logical interpretation of the scalar term *some*, whereas the majority of the five-year-olds favors a pragmatic interpretation. This difference was also clear from the four control sentences that could be answered logically in the ABT. About half the time, the three-year-olds spontaneously produced a logical answer, whereas the five-year-olds practically never did.

We made a distinction between two tasks because we expected, in accordance with Pouscoulous et al. (2007), the ABT to lead to more pragmatic answers than the TVJT. We only found a reliable difference between the two tasks regarding the number of correct answers on the control sentences. As expected, the ABT was easier than the TVJT, but it did not lead to significantly more pragmatic answers.

Because we found evidence that pragmatic competence increases with age, this indirectly supports the assumption that pragmatic reasoning requires cognitive resources. As Pouscoulous et al. (2007) suggested, cognitive resources are important in implicature production and may explain why easier tasks, that require less cognitive resources, lead to more pragmatic answers than more difficult tasks. In adults, it has been shown that burdening WM decreases implicature production by 10 per cent (De Neys & Schaeken, 2007). Moreover, Dieussaert, Verkerk, Gillard, and Schaeken (2011) found an interaction between cognitive load and WM capacity that influences pragmatic reasoning. They measured participants’ WM capacity by means of the Operation Span Task for group testing and created three WM groups based on the performance on this WM task: the low-, middle- and high span group. They found an effect of cognitive load, only for the participants with a low WM capacity. The low span group provided fewer pragmatic answers when WM was burdened with a secondary task. The middle- and high span groups’ pragmatic answering was not influenced by the cognitive load. This finding, that especially low capacity people are influenced by cognitive load, leads to the assumption that an effect of WM should be found in children’s pragmatic reasoning because children’s cognitive resources are limited. So far, no research has been conducted on children that directly investigated the role of cognitive resources.

Based on the findings of De Neys and Schaeken (2007) and Dieussaert et al. (2011), it can be assumed that people with less cognitive resources will be less pragmatic than people with more cognitive resources. In Experiment 2 we will measure WM capacity in five-year-old children and investigate whether children with a high WM capacity produce more scalar implicatures than children with a low WM capacity.

## Experiment 2

### Method

#### Participants

The sample comprised 48 five-year-olds (28 boys and 20 girls) between the ages of 62 and 73 months with a mean age of 67 months (*SD*=2.86), recruited from two different schools in Belgium. None of these children participated in Experiment 1. All were native Dutch speakers.

#### TVJT, ABT

The same TVJT and ABT were used as in Experiment 1.

#### Working Memory Tasks

The children performed three WM tasks. First, the auditory (phonological loop) component was measured using the Digit Span Forward task in which participants have to repeat an orally presented list of numbers. The list starts with a sequence of two numbers and keeps increasing until the child makes two errors within one block of the same digit length. Second, the visual component (visuo-spatial sketchpad) was measured using the Corsi Block Span test. In this test, the children were presented with nine wooden blocks on which the experimenter tapped a pattern and the children were instructed to repeat the sequence. The sequence becomes longer until the child makes two errors within one block of the same difficulty level. The third WM task, which was intended to provide a ‘central executive’ measure, was the Digit Span Backward task. This task is identical to the Digit Span Forward, except that the participant needs to repeat the sequence of numbers in reverse order. The raw scores for each of these tasks (i.e. the total number of correct answers) were converted into z-scores, which were then added up to compute the WM span.

#### Procedure

The procedure was exactly the same as in Experiment 1. The only difference was the extra WM measure. All children first completed the three WM tasks and next, the order of the other two tasks was randomized, so that half of the participants started with the TVJT and the other half with the ABT.

### Results

As in Experiment 1, we first controlled whether we could exclude an effect of order of the two implicature tasks on pragmatic responding. We found no significant effect of order, nor on the ABT (*t*(46)=-1.65; *p*=.11), nor on the TVJT (*t*(46)=-0.19; *p*=.85).

Even though we did not find a significant difference between the ABT and the TVJT in Experiment 1, we hypothesized that there would be differences in implicature production and performance between the TVJT and the ABT. Our second hypothesis concerned an effect of WM.

Our first hypothesis about the difference in performance was confirmed by the finding that the TVJT leads to significantly more errors than the ABT on the control statements (8.5 per cent versus 1.5 per cent, respectively; Wilcoxon Signed Ranks test, *n*=26; *T*=20.5; *p*<.001).

With regard to the critical sentences, we hypothesized that the ABT would lead to more pragmatic answers than the TVJT. Again, our hypothesis was confirmed. The children responded pragmatically to the critical sentences in 90.5 per cent of the instances on the ABT, compared to 70.0 per cent on the TVJT (Wilcoxon Signed Ranks test, *n*=20; *T*=22.5; *p*=.001). When the affirmative and the negative critical item are considered separately, we see that the pragmatic response rate is lower for the negative item than for the affirmative item on both the TVJT and the ABT. However, these differences were not significant (TVJT: *t*(47)=2.001, *p*=.051; ABT: *t*(47)=1.944, *p*=.058).

With respect to our second hypothesis concerning a WM effect, we performed a tertile split based on the children’s WM span (low span group: *N*=16; *M*=-2.37; *SD*=1.42; middle span group: *N*=16; *M*=0.29; *SD*=0.60; high span group: *N*=16; *M*=2.13; *SD*=0.82). In our analyses we compared the highest WM span group with the lowest WM span group in order to maximize the difference in WM span between the two groups. The two span groups were compared with regard to the number of correct answers on the control sentences and the number of pragmatic responses, for each of the two tasks. The results of all children are displayed in Table 1,^1^.

**Table 1:** Percentages correct answers on the control sentences and percentages pragmatic answers on the critical sentences of the ABT and TVJT for low-, middle- and high WM span children (Experiment 2).

	Control Sentences			Critical Sentences		

	*Low span (n=16)*	*Middle span (n=16)*	*High span (n=16)*	*Low span (n=16)*	*Middle span (n=16)*	*High span (n=16)*

TVJT	87.5	93.1	93.8	56.3	78.1	75.0
ABT	95.6	100.0	100.0	93.8	90.6	87.5

There were no significant differences in pragmatic processing between the high- and the low span group, not even when we looked at the affirmative and negative critical items separately for each of the two tasks. However, the number of correct responses to the unambiguous control sentences differed significantly between the two groups. The high span group was more accurate than the low span group on both the ABT (100 per cent vs 95.6 per cent correct answers; Mann-Whitney U test, *n_1_*=16, *n_2_*=16; *U*=96; *Z*=-2.099; *p*=.018) and the TVJT (93.8 per cent vs 87.5 per cent correct answers; Mann-Whitney U test, *n_1_*=16, *n_2_*=16; *U*=76; *Z*=-2.079; *p*=.019).

When we look at the number of logical answers on the control sentences of the ABT, we found that the low span group produced more logical answers than the high span group (10.9 per cent vs 4.7 per cent). However, this difference was not significant (*X*^2^=1.74, *df*=1, *p*=.188).

### Discussion

In contrast to Experiment 1, the ABT did lead to significantly more pragmatic answers than the TVJT in Experiment 2. In addition, the five-year-olds made fewer mistakes on the ABT control statements than on the TVJT control statements. These results indicate that metalinguistic tasks are harder than tasks that don’t require a verbal response. A possible reason why the difference in pragmatic reasoning was not found for the five-year-olds in Experiment 1 could be that the sample of children was too small.

The results of Experiment 2 show that five-year-old children are competent pragmatic reasoners. Their competence is still ‘vulnerable’, but taking into account certain factors such as task complexity, task content etc., they are capable of producing scalar implicatures on a high level. This confirms the findings of Pouscoulous et al. (2007). Moreover, the validity of our results was enhanced by manipulating the nature of the task within participants and by changing the design of the TVJT to make it more comparable to the ABT. This allows us to attribute the results to the task’s cognitive demands and to conclude that the nature of the task is very important in implicature processing in five-year-olds.

Our WM measures revealed no significant differences in implicature processing between a group of low span children and a group of high span children. The high span children made significantly fewer errors on the control statements of both tasks and were less logical on the control statements of the ABT (although this difference was not significant). Even so, these WM results do not allow us to draw firm conclusions about the role of WM in implicature processing.

Remarkably, the five-year-olds in our experiments produced a much higher percentage of pragmatic answers than the children tested in Pouscoulous et al. (2007). They were equally pragmatic on the ABT and more pragmatic on the TVJT than the seven-year-olds and the adults in Pouscoulous et al. (2007), who concluded that ‘Only 7-year-olds reveal behavior that approaches that of adults among the standard cases and even among them adultlike implicature performance is less likely when it concerns negative sentences’ (Pouscoulous et al., 2007: 371).

Since the age of seven is mostly found to be the age at which children really begin to demonstrate pragmatic skills (e.g., Guasti et al., 2005), we ran the same experiment with a group of seven-year-olds. We expected them to be even more pragmatic than the five-year-olds. In addition to the ABT and TVJT used in Experiment 1 and Experiment 2, we included a TVJT that is often used in experimental research on implicatures, i.e. the world-knowledge TVJT from Noveck (2001). By including this task, the children have to perform two different TVJT’s that only differ in the specific content used. The TVJT that was also used in Experiment 1 and Experiment 2 involves simple materials (marbles and boxes) while the content of the other TVJT requires children to rely on their knowledge of the world. We expect this to be more difficult than the other TVJT.

Even though we did not find a significant WM effect among the five-year-olds of Experiment 2, we also measured WM in the seven-year-olds in Experiment 3. The WM tasks used in Experiment 2 were originally designed for children from the age of six (Working Memory Test Battery for Children (WMTB-C); Pickering & Gathercole, 2001). This means that the absence of a reliable WM effect might be attributed to the difficulty of the WM tasks that were used. These tasks should be suitable for seven-year-olds.

## Experiment 3

### Method

#### Participants

Thirty-four seven-year-olds (18 girls, 16 boys) between the ages of 6.9 and 8.5 with a mean age of 7.5 (*SD*=0.32) participated in this experiment. All participants were recruited from the same school and were native Dutch speakers.

#### TVJT, ABT and WM Tasks

The same TVJT, ABT and three WM tasks were used as in Experiment 2.

#### World-knowledge TVJT

In order to investigate whether the specific content of the task plays a role in implicature production, the seven-year-olds conducted a task based on Noveck (2001; Experiment 3). In this task, the children were presented with 30 statements (translated into Dutch) and were instructed to indicate whether or not they agreed with each statement. The sentences were based on three types of information: factually universal, factually existential and absurd. The statements can be categorized in six subgroups:

- Five absurd *all* sentences (e.g., *all* birds have telephones.)
- Five absurd *some* sentences (e.g., *some* fish are made of leaves.)
- Five true *all* sentences (e.g., *all* elephants have trunks.)
- Five true (and felicitous) *some* sentences (e.g., *some* flowers are yellow.)
- Five false *all* sentences (e.g., *all* dogs have spots.)
- Five true (but pragmatically infelicitous) *some* sentences (e.g., *some* giraffes have long necks.)

We were particularly interested in the sentences from category (f). If children agree with such statements they are responding logically, while disagreeing implies a pragmatic response. If we look at the different types of statements, it is clear that switching quantifiers can make (c) interchangeable with (f) as well as (d) with (e). In this way, we created two versions of this task. In each version, both the *all* and the *some* sentences were randomized, as were the different types of statements.

#### Procedure

The procedure was exactly the same as in Experiment 2. However, an additional test was administered after all other tasks were performed. All children received a paper with the 30 statements included in the world-knowledge TVJT. These statements were read out to them and they were asked to indicate, for each statement, whether they agreed or disagreed by circling the appropriate answer.

### Results

We first controlled whether we could exclude an effect of order of the implicature tasks on the pragmatic response rate. We found no significant effect of order, not on the ABT (*t*(32)=0; *p*=1), nor on the TVJT (*t*(32)=-1.84; *p*=.076).

We had two different hypotheses. The first hypothesis concerned an effect of the nature of the task. We expected the ABT to be easier than the TVJT that, in turn, was expected to be easier than the world-knowledge TVJT. Accordingly, we expected the ABT to lead to the most pragmatic answers and the world-knowledge TVJT to the least. Our second hypothesis concerned an effect of WM.

Regarding our first hypothesis, the TVJT control statements led to 95.9 per cent correct answers, compared to 100.0 per cent for the ABT (Wilcoxon Signed Ranks test, *n*=13, *T*=91.0, *p*<.001). For the control statements of the world-knowledge TVJT, the number of correct answers was 94.0 per cent which differed significantly from the ABT (Wilcoxon Signed Ranks test, *n*=25, *T*=325.0, *p*<.001) and marginally significantly from the other TVJT (Wilcoxon Signed Ranks test, *n*=28, *T*=133, *p*=.055). Regarding the critical sentences, there were no significant differences between the TVJT and the ABT in the number of pragmatic answers (91.2 per cent versus 94.1 per cent, respectively; Wilcoxon Signed Ranks test, *n*=8, *T*=22.5, *p*=.24). In contrast, the world-knowledge TVJT only yielded 69.4 per cent pragmatic answers, which differed significantly from the other TVJT (Wilcoxon Signed Ranks test, *n*=23, *T*=229.5, *p*=.003) and from the ABT (Wilcoxon Signed Ranks test, *n*=22, *T*=34.5, *p*=.002).

As in Experiments 1 and 2, we also looked at the affirmative and the negative critical item of the TVJT and the ABT separately (there was no negative critical item in the world-knowledge TVJT). There was no significant difference in the pragmatic response rate between the affirmative and the negative critical item of the ABT (*t*(33)=1.00; *p*=.33) but there was a significant difference for the TVJT (*t*(33)=2.659; *p*=.012). The affirmative critical item of the TVJT led to 100 per cent pragmatic answers compared to 82.4 per cent for the negative critical item.

Regarding our second hypothesis, we performed a tertile split as in Experiment 2 (low span group: *N*=11; *M*=-2.38; *SD*=1.06; middle span group: *N*=12; *M*=0.06; *SD*=0.67; high span group: *N*=11; *M*=2.32; *SD*=1.07). Again, in our analyses we only compared the group of high WM span children with the low span group. The results of all children are displayed in Table 2. No significant differences were found between the high- and low span group on any of the three tasks, neither in pragmatic responses, nor in performance on the unambiguous control sentences.^2^ These differences were also non-significant when we looked at each critical sentence of the ABT and the TVJT separately.

**Table 2:** Percentages correct answers on the control sentences and percentages pragmatic answers on the critical sentences of the ABT, TVJT and world-knowledge TVJT for low-, middle- and high WM span children (Experiment 3).

	Control Sentences			Critical Sentences		

	*Low span (n=11)*	*Middle span (n=12)*	*High span (n=11)*	*Low span (n=11)*	*Middle span (n=12)*	*High span (n=11)*

TVJT	96.4	95.8	95.5	100	87.5	86.4
ABT	100.0	100.0	100.0	95.5	87.5	100
World-knowledge TVJT	92.8	94.3	94.9	67.3	58.3	83.6

## General Discussion

The three experiments reported in this article investigated pragmatic competence in young children. In Experiment 1, both three-year-olds and five-year-olds performed a metalinguistic TVJT and an ABT, in which children did not have to answer verbally. Children as young as three years had never been investigated in scalar implicature research. Our results showed that five-year-olds are competent pragmatic reasoners who interpret *some* mostly pragmatically, whereas three-year-olds equally adhere to the logical and the pragmatic meaning of *some*. This indicates lack of pragmatic competence since their performance on the control sentences revealed overall linguistic competence with the quantors used in the tasks. The three-year-olds’ logical interpretation of *some* was also shown in the control sentences of the ABT that could be answered logically. The three-year-olds spontaneously provided the logical answer significantly more than the five-year-olds.

Contrary to our expectations, Experiment 1 revealed no difference in the number of pragmatic answers between the two different tasks. Based on the findings of Pouscoulous et al. (2007), we expected the ABT to be easier than the TVJT and therefore to lead to more pragmatic answers. We did find a significant difference in the difficulty of the task (the control sentences of the ABT were answered more accurately than the control sentences of the TVJT) but the ABT did not lead to significantly more pragmatic answers than the TVJT.

In Experiment 2, a group of five-year-olds performed the same tasks as in Experiment 1. Additionally, a measure of WM was included. Based on the assumption that pragmatic reasoning requires cognitive effort, we expected an effect of WM. We expected children with a high WM capacity to be more pragmatic than children with a low WM capacity since they have more cognitive resources available. As in Experiment 1, we also wanted to test the hypothesis that the nature of the task plays an important role in implicature research. In contrast to Experiment 1, this hypothesis was confirmed in Experiment 2. We found, as expected, that a more difficult TVJT caused the children to be less accurate and less pragmatic than an ABT in which children did not have to answer verbally. This difference cannot be caused by a difference in task design -because the two tasks were similar in design- but by a difference in task complexity. Manipulating the nature of the task is sufficient to show that, under the right circumstances, children as young as five years are capable of spontaneously producing implicatures. It is unclear why we did not find an effect of the nature of the task in Experiment 1. Since the three-year-olds showed very little pragmatic competence, we should look only to the group of five-year-olds. However, even if we only consider the group of five-year-olds in Experiment 1, no effect of the nature of the task can be found. It might be that the sample of five-year-olds was too small to find a significant effect.

We did not find any support for our hypothesis concerning WM. Five-year-olds with a high WM capacity were not significantly more pragmatic than those with a low WM capacity.

In Experiment 3, we investigated a group of seven-year-olds whom we expected to be even more pragmatic than the five-year-olds in Experiment 2. They performed the same tasks as in Experiment 2, including the WM tasks. Additionally, an extra task was administered: a TVJT based on world-knowledge that is often used in scalar implicature research (e.g., Noveck, 2001).

The expectation that seven-year-olds would provide even higher rates of pragmatic answers than five-year-olds was confirmed: the pragmatic response rate was so high that it did not lead to a significant difference between the ABT and the TVJT. However, when the children performed a TVJT involving world-knowledge statements, pragmatic responses dropped by 22 per cent. For the world-knowledge TVJT, the children needed to rely on the knowledge they have stored in their memory, whereas in the simple TVJT, they just had to rely on the boxes and marbles in front of them, which is less demanding on memory resources. Another difference between the two TVJT’s that might influence pragmatic reasoning is that the TVJT with the marbles and the boxes was based on visual input (the marbles and the boxes) whereas the world-knowledge TVJT was not based on visual input.

This difference in the number of pragmatic answers between the two TVJT’s shows that not only the nature of the task plays an important role in scalar implicature processing, but also the specific task content. The instructions of the two tasks were completely identical –indicating whether statements are wrong or right- but the content of the statements differed. The more cognitive world-based knowledge was required, the less pragmatic answers were provided. This cognitive content specifically affected pragmatic processing since the seven-year-olds proved to possess the world-knowledge required to judge the statements correctly by being highly accurate on the control statements of the world-knowledge TVJT.

The hypothesis that easier tasks lead to significantly more pragmatic answers than more difficult tasks is based on the assumption that cognitive resources are critical in implicature production (De Neys & Schaeken, 2007). As easier tasks require fewer cognitive resources than complex tasks, more cognitive resources remain available for producing implicatures. However, similar to Experiment 2, we did not find a reliable WM effect in the seven-year-olds. We did find that the high span children were more pragmatic than the low span children on the most difficult task in each experiment (the TVJT in Experiment 2 and the world-knowledge TVJT in Experiment 3). Although this trend can be observed in our WM data, we were unable to find a single significant WM effect. However, it is worth mentioning that even though we would have expected a WM effect, the absence of a reliable effect is not that surprising given that the significant WM effect found in adults was only small (De Neys & Schaeken, 2007). In order for communication to go smoothly, this cannot require too much working memory.

A possibility for future research is to manipulate WM the same way De Neys and Schaeken (2007) did. Instead of measuring WM they presented participants with a secondary task in order to burden WM. A secondary task based on De Neys and Schaeken (2007), adapted for child use, might be a better method to investigate the role of WM in implicature production in children.

A specific issue we controlled for in our analyses was the difference between affirmative and negative critical statements. Because negation statements should be cognitively more demanding, we expected to find more logical answers on the negative items than on the affirmative items. However, in Experiment 1 we found no difference between the two critical items of the ABT and the significant difference between the critical items of the TVJT was opposite to our expectation. The children’s pragmatic response rate was higher on the negative item than on the affirmative item. This difference was caused by an unexplainably low number of pragmatic answers provided by the three-year-olds on the affirmative item. We can not think of an obvious cause that can explain this observation.

In Experiment 2, there was no significant difference in pragmatic response rate between the affirmative and negative item, neither on the TVJT, nor on the ABT.

Finally, in Experiment 3, the difference between the two critical items was only significant for the TVJT. In this case, the difference was in line with our expectation. The affirmative item evoked significantly more pragmatic answers than the negative item. However, when we take the results of all three experiments together, there is no clear pattern that negation statements are significantly harder than the affirmative statements and therefore lead to a higher number of pragmatic answers.

In sum, in three experiments we replicated the finding that there is a clear developmental trend in pragmatic competence. Three-year-olds show little pragmatic competence compared to five-year-olds and especially seven-year-olds who clearly understand and prefer the pragmatic meaning of *some*. A second important finding is that the nature of the task and the specific task content are very important in scalar implicature production in young children: more cognitive tasks or more cognitive task content cause a decrease in implicature production. It is important that this factor is taken into account when investigating implicature production in children because it can lead to wrongly drawn conclusions. Another factor that might need to be taken into account in future research is a measure of general language ability. Since it was found that metalinguistic tasks are harder than action tasks, it is plausible that general language ability may account at least partly for these results. It could be that, for such young children, general language ability is more important than WM capacity.

Finally, it is worth mentioning that pragmatic competence seems inextricably linked to people’s mother language. Pouscoulous et al. (2007) had already shown that there is a difference in the number of pragmatic answers between the French *certains* and *quelques*, used as translations of *some*. Likewise, compared to other developmental implicature studies, it seems that Dutch speaking children are highly pragmatic when interpreting *some*.

## Footnotes

We also performed analyses on the whole sample of children (in contrast to only the high- and the low load children). In these analyses WM score was included as a continuous variable. The correlations between WM score and the pragmatic response rate were not significant for both the TVJT and the ABT. However, the correlations between WM score and accuracy on the control sentences were significant for both the TVJT (*r*=0.33; *p*=.021) and the ABT (*r*=0.326; *p*=.024).

The analyses on the whole sample of children revealed that there were no significant correlations between WM score and pragmatic response rate, nor between WM score and accuracy on the control sentences, for all three tasks.
